# Construction and optimization of a biocatalytic route for the synthesis of neomenthylamine from menthone

**DOI:** 10.1186/s40643-023-00693-w

**Published:** 2023-11-03

**Authors:** Hui-Jue Zhu, Jiang Pan, Chun-Xiu Li, Fei-Fei Chen, Jian-He Xu

**Affiliations:** Laboratory of Biocatalysis and Synthetic Biotechnology, State Key Laboratory of Bioreactor Engineering, Shanghai Collaborative Innovation Centre for Biomanufacturing, College of Biotechnology, East China University of Science and Technology, Shanghai, 200237 People’s Republic of China

**Keywords:** Biocatalysis, (+)-Neomenthylamine, Transaminase, Asymmetric synthesis, (+)-*N*-Boc-Neomenthylamine

## Abstract

(+)-Neomenthylamine is an important industrial precursor used to synthesize high value-added chemicals. Here, we report a novel biocatalytic route to synthesize (+)-neomenthylamine by amination of readily available (−)-menthone substrate using ω-transaminase. By screening a panel of ω-transaminases, an ω-transaminase from *Vibrio fluvialis* JS17 was identified with considerable amination activity to (−)-menthone, and then characterization of enzymatic properties was conducted for the enzyme. Under optimized conditions, 10 mM (−)-menthone was transformed in a mild aqueous phase with 4.7 mM product yielded in 24 h. The biocatalytic route using inexpensive starting materials (ketone substrate and amino donor) and mild reaction conditions represents an easy and green approach for (+)-neomenthylamine synthesis. This method underscores the potential of biocatalysts in the synthesis of unnatural terpenoid amine derivatives.

**Graphical Abstract:**

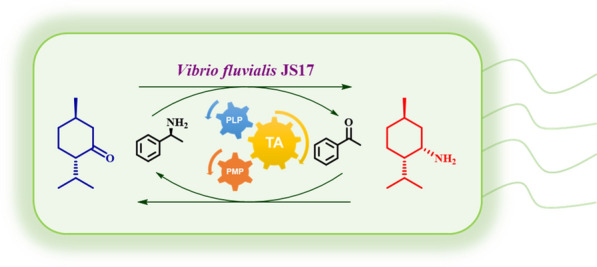

**Supplementary Information:**

The online version contains supplementary material available at 10.1186/s40643-023-00693-w.

## Introduction

**Scheme 1:**
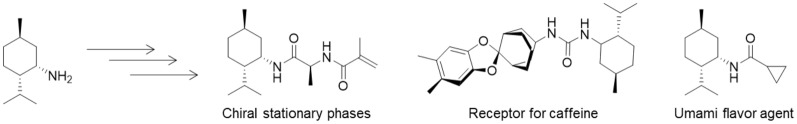
Commercial applications of (+)-neomenthylamine derivatives

Enantiomerically amines are a significant class of intermediates for the synthesis of pharmaceuticals, agrochemicals, and fine chemicals (Shin and Kim 1999; Fuchs et al. 2010; Ghislieri and Turner 2014; Costa et al. 2017). Optically pure (+)-neomenthylamine has been utilized as a building block for chiral stationary phase to resolute enantiomers in HPLC (Arlt et al. 1991). Some (+)-neomenthylamine derivatives were used as great umami flavor agents (Welschoff and Waldvogel 2010) (Scheme 1).

Currently, all the synthetic pathways of (+)-neomenthylamine rely on chemical methods with terpenoid initial reactants such as (−)-menthone (Wallach 1891; Kozlov et al. 1981; Kozlov 1982). A conventional way was to convert (−)-menthone by reductive amination under Leuckart–Wallach conditions (Kitamura et al. 2002), which could obtain all the neomenthylamines isomers. An alternative approach was to reduce menthone oxime to amines under Bouveault–Blanc conditions using an excess equivalent of sodium (Han et al. 2017). Besides, dielectrically controlled resolution (DCR) of racemic neomenthol by (*R*,*R*)-tartaric acid and electrochemical synthesis with mercury pool cathode were both used to produce optically pure (+)-neomenthylamine stereoselectively (Kulisch et al. 2011; Schmitt et al. 2014; Reinscheid and Reinscheid 2016). In recent reports, hydrogenation of menthone oxime to amines via gold catalysts could afford excellent yields at 100 °C. The drawbacks of this system are the high cost of gold nanoparticles and use of high temperature (Demidova et al. 2020).

Over the past 2 decades, biocatalysts are increasingly being employed for the synthesis of chiral amines due to their mild reaction conditions, high chemo/regio-selectivity, and atom economy advantages (Mayol et al. 2016; López-Iglesias et al. 2016; Wu and Li 2018; Hwang and Lee 2019; Cao et al. 2022; Wu et al. 2022). A remarkable example of industrial application is in the production process of the antidiabetic compound sitagliptin using an (*R*)-selective transaminase from *Arthrobacter* sp., which brought Merck about 6 billion U.S. dollars of revenue in 2016 (Savile et al. 2010; Ferrandi and Monti 2018).

**Scheme 2:**
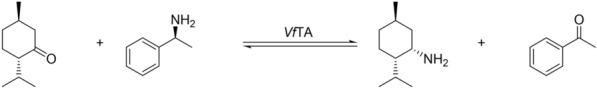
Synthesis of (+)-neomenthylamine via ω-transaminase *Vf*TA-mediated amination of (−)-menthone

In this study, we develop a simple and green biocatalytic route to synthesize (+)-neomenthylamine. The reaction is achieved by amination of (−)-menthone with a newly screened ω-transaminase from *Vibrio fluvialis* JS17 using (*S*)-α-methylbenzylamine (*S*-MBA) as amino donor in a mild aqueous phase (Scheme 2). This biocatalytic approach features apparent sustainability advantages, including avoidance of costly and poisonous catalysts, mild temperature and pressure operating conditions, easy product separation and low environmental impact, which is generally unmatched for traditional chemical methods.

## Materials and methods

### Materials

(−)-Menthone was purchased from Meryer Chemical Technology Co., Ltd. (Shanghai, China). All other reagents of analytical grade were obtained from commercial sources. Yeast extract and tryptone were purchased from Oxoid (Hampshire, UK). Genes were synthesized by Generay Biotech Co., Ltd. (Shanghai, China). All transaminases, amine dehydrogenases, imine reductases, reductive aminases, and aminodeoxychorismate lyases were obtained from the enzyme libraries saved in our laboratory.

### Gene cloning and expression

All genes were cloned into the pET-28a (+) vector and transformed into the *E. coli* BL21 (DE3) cells. Positive transformants and strains preserved in glycerol stock were grown in Luria Broth media containing 50 μg/mL kanamycin overnight in a 37 °C incubator. Subsequently, the suspensions were transferred into Terrific Broth media for incubation until OD_600_ reached to 0.6. Protein overexpression was induced by adding 0.2 mM isopropyl-d-thiogalactopyranoside (IPTG) followed by incubation for 24 h at 16 °C and 220 rpm. Cells were harvested by centrifugation at 12,000×*g*. All cell pellets were stored at − 20 °C.

### General screening

Screening reactions were carried out at 1 mL scale in Eppendorf tubes. Reaction I for the screening of amine dehydrogenases: 5 mM (−)-menthone, 1 mM NAD^+^, 1.5 mg/mL *Cb*FDH, 0.2 g/mL AmDHs lysates in 5 M NH_4_OH/NH_4_COOH buffer (pH 9.0). Reaction II for the screening of transaminases and aminodeoxychorismate lyases: 5 mM (−)-menthone, 50 mM DL-Ala or isopropylamine or 2-pentanamine, 0.5 mM pyridoxal-5′-phosphate (PLP), 15 g_cdw_/L TAs or ADCLs whole cells, 2% (v/v) DMSO in 100 mM KPB buffer (pH 8.0). Reaction III for the screening of imine reductases and reductive aminases: 5 mM (−)-menthone, 5 mM isopropylamine, 1 mM NADP^+^, 30 mM D-Glu, 3 mU/mg *Bm*GDH, 0.2 g/mL IREDs or RedAms lysates in 100 mM KPB (pH 7.0). All the reactions were mixed in a high-speed shaker at 30 °C and 1000 rpm (Allsheng Instruments Co., Ltd., Hangzhou, China) and analyzed by GC–MS.

GC–MS analysis was performed on Shimadzu-QP2010 gas chromatography–mass spectrometry equipped with a Rxi®-5Sil MS column (30 m × 0.25 mm, 0.25 μm) (injector and detector temperatures at 250 °C, oven temperature at 80 °C, ion source temperature at 230 °C, split ratio at 30:1). For analysis, the injection volume was 1 μL and the temperature of column was initially set as 80 ℃, raised to 230 ℃ at 2 ℃ min^−1^ and held for 2 min.

### Protein purification

Chromatography was performed on a Ni–NTA His-trap column with the following buffers: buffer A: 50 mM Tris, 500 mM NaCl, 10 mM imidazole, 0.375% (v/v) *β*-mercaptoethanol, pH 7.0; buffer B: 50 mM Tris, 500 mM NaCl, 500 mM imidazole, 0.375% (v/v) *β*-mercaptoethanol, pH 7.0; buffer C: 50 mM Tris, 150 mM NaCl, 1 mM DTT, 25% (v/v) glycerin, pH 7.0. All cells were resuspended and filtered through a 0.45-µm low protein binding membrane. The column was equilibrated with 10 column volumes of buffer A. Subsequently, enzymes were eluted with 75% buffer B after removal of non-specifically bound proteins with 25% buffer B. The purified enzymes were collected by buffer C and stored at −80 °C.

### Activity assay

Enzyme activity assays of *Vf*TA were performed at 30 °C for 20 min in potassium phosphate buffer (pH 6.0) containing 5 mM (−)-menthone as substrate, 30 mM *S*-MBA as amino donor, 2 mM PLP, and 1.5 mg/mL purified enzyme. Reactions were quenched with 10 M NaOH solution, extracted with methyl tert-butyl ether (MTBE) and analyzed by GC. Kinetic parameters were determined for the amination of (−)-menthone with *Vf*TA. Assay mixtures containing (0–50 mM) (−)-menthone, 60 mM *S*-MBA, 2 mM PLP, 2mg/mL purified *Vf*TA in potassium phosphate buffer (pH 6.0) were incubated at 30 °C for 20 min and quenched with 10 M NaOH solution. The quenched samples were extracted with MTBE and analyzed by GC for (+)-neomenthylamine. Similar conditions were used to determine kinetic parameters for *S*-MBA with *Vf*TA. All data were subjected to nonlinear regression fitting in GraphPad Prism 9.0. or Origin 2018 to obtain kinetic parameters.

GC analysis was performed on Shimadzu-2014 gas chromatography equipped with a DB-1701 column (30 m × 0.25 mm, 0.25 μm) (injector temperature: 250 °C, split ratio: 30:1). For analysis, the injection volume was 1 μL and the temperature of column was initially set as 50 ℃, raised to 90 ℃ at 10 ℃ min^−1^, and then increased to 260 ℃ at 20 ℃ min^−1^.

### Thermostability assay

Half-life and melting temperature (*T*_m_) assays of *Vf*TA were determined in this study. The purified *Vf*TA was diluted to 2 mg/mL with buffer C and incubated at 30 °C, 40 °C, and 50 °C, respectively. Samples were extracted at regular intervals to calculate residual activity through GC analysis.

Data were subjected to first-order inactivation kinetic equation for nonlinear regression fitting to obtain *t*_1/2_ in Origin 2018. *T*_m_ assays of *Vf*TA were determined by detecting the CD absorbance values in the wavelength range of 180–260 nm at various temperatures using a circular dichroism spectrometer (Applied Photophysics Ltd., Oxford, UK). Data were analyzed with Global 3 analysis to determine *T*_m_ values.

### Bioinformatics

MEGA 11 was used to perform multiple sequence alignment. Phylogenetic trees were built with the neighbor-joining algorithm within the Molecular Evolutionary Genetics Analysis Version 11 (MEGA 11) and present the relative positions of the proteins labeled with species name (Lewis et al. 1995). Molecular modeling for this project was performed using crystal structures of *Vibrio fluvialis* JS17 (PDB:5ZTX) and various ligands were docked by AutoDock Vina program package. The center grid box was defined by x, y, z coordinates of − 1.437, − 5.754, and 20.424, respectively, with a volume size of 15*15*15.

### Scale-up protocol

A preparative-scale mixture with a volume of 300 mL, containing 10 mM (−)-menthone, 60 mM *S*-MBA, 2 mM PLP, and 100 g/L *Vf*TA whole cells in potassium phosphate buffer (pH 6.0) was incubated at 30 °C and 200 rpm for 28 h. The reaction was stopped by adding 15 mL 10 M NaOH solution and extracted with MTBE (3 × 300 mL). The organic extract was dried over anhydrous Na_2_SO_4_ and concentrated under reduced pressure vacuum to obtain (+)-neomenthylamine product.

### Boc derivatization

Mixture of 5 mmol (+)-neomenthylamine, 7.5 mmol di-tert-butyl-dicarbonate, and 5 mmol TEA was dissolved in 500 mL DCM. The reaction was maintained at room temperature with reflux using a condenser for 4 h. Reaction mixture after concentration was separated on silica gel column with PE/EA = 200/1 (v/v). The organic phase was concentrated under reduced pressure to obtain (+)-Boc-neomenthylamine product.

## Results and discussion

### Discovery of an ω-transaminase for amination of (−)-menthone to (+)-neomenthylamine

**Fig. 1:**
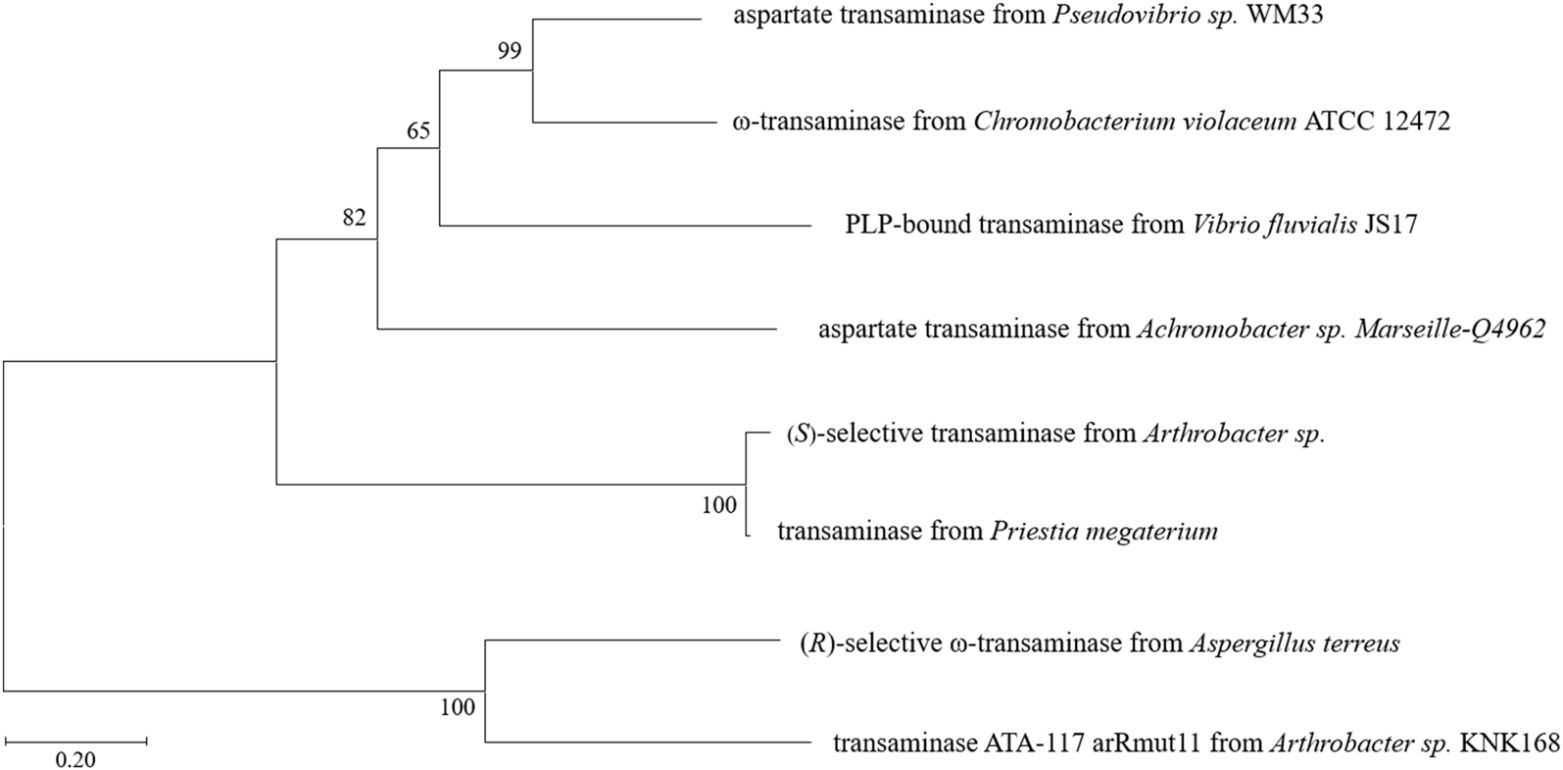
Phylogenetic analysis of the *Vf*TA and the homologous proteins

Since the enzymatic reaction pathway from (−)-menthone to (+)-neomenthylamine has not been reported yet, we dissected the chemical structure of (−)-menthone and observed that it features a cyclohexane skeleton with a carbonyl group and alkyl substitutions. Leveraging this characteristic, we devised a substrate analog screening strategy, employing the amination of cyclohexanone or its analogs as a model reaction to discover enzymes that can convert (−)-menthone to (+)-neomenthylamine. Subsequently, we screened 83 target enzymes from different sources capable of catalyzing amination reaction, including transaminases (TAs), imine reductases (IREDs), reductive amine enzymes (RedAms), amine dehydrogenases (AmDHs), and aminodeoxychorismate lyases (ADCLs) (Koszelewski et al. 2011; Tufvesson et al. 2012; Skalden et al. 2015; Wetzl et al. 2016; Guo and Berglund 2017; Ramsden et al. 2019; Gavin et al. 2019; Mangas-Sanchez et al. 2020). After expressing and rescreening of the enzymes in the form of cell-free extract, we obtained a transaminase (*Vf*TA) from *Vibrio fluvialis* JS17 (PDB: 5ZTX) that can convert (−)-menthone into (+)-neomenthylamine successfully. A phylogenetic analysis was constructed to analyze the evolutionary relationship of *Vf*TA with the previously reported ω-transaminases that catalyze the amination of cyclohexanone and its analogs (Fig. 1). As a result, *Vf*TA was situated at the same clade with the transaminases from *Pseudovibrio* sp. WM33 and *Chromobacterium violaceum* ATCC 12472, demonstrating a relatively close evolutionary relationship with the two enzymes.

Different amino donors were then investigated for *Vf*TA-catalyzed amination of menthone, and interestingly, *Vf*TA exhibited different activities and selectivities for different amino donors (Table 1). Compared with DL-alanine and 2-pentanamine, using isopropylamine as the amino donor resulted in the highest yield of (+)-neomenthylamine by *Vf*TA, reaching 0.19 mM with a conversion rate of 3.8%.

**Table 1:** Asymmetric synthesis of (+)-neomenthylamine using *Vf*TA with different amino donors

Enzyme	DL-Ala		Isopropylamine		2-Pentanamine	
	Conv. (%)^a^	*d.e.* (%)^b^	Conv. (%)^a^	*d.e.* (%)^b^	Conv. (%)^a^	*d.e.* (%)^b^
*Vf*TA	0.9	79 (3*S*, 4*S*)	3.8	82 (3*S*, 4*S*)	2.1	80 (3*S*, 4*S*)

^a^Conversion and the value of diastereomeric excess was determined by GC–MS on an achiral phase. The screening reaction system (1 mL) consists of potassium salt phosphate buffer (100 mM KPB pH 8.0), 5 mM (−)-menthone, 50 mM DL-Ala or isopropylamine or 2-pentanamine, 0.5 mM pyridoxal-5′-phosphate (PLP), 15 g_cdw_ L^−1^ whole cells, 10% (v/v) dimethyl sulfoxide. All the reaction mixtures were shaken for 24 h at 30 °C and 1000 rpm in a high-speed shaker

^b^ *D.e.* (%) = $$|\frac{\left[\left(3{\text{S}}, 4{\text{S}}\right)+(3{\text{R}}, 4{\text{S}})\right]-[\left(3{\text{S}}, {\text{R}}\right)+(3{\text{R}}, 4{\text{R}})]}{\left[\left(3{\text{S}}, 4{\text{S}}\right)+(3{\text{R}}, 4{\text{S}})\right]+\left[\left({3}{\text{S}}, 4{\text{R}}\right)+(3{\text{R}}, 4{\text{R}})\right]}|$$

### Optimization of system parameters for *Vf*TA-mediated transamination reaction

**Fig. 2:**
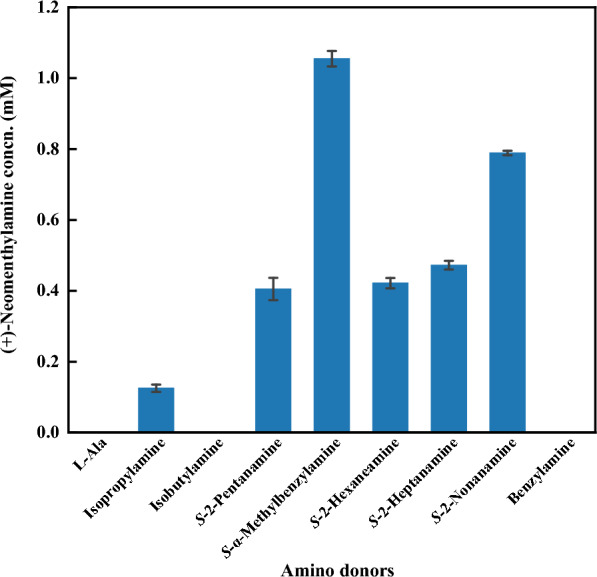
Effect of amino donors of *Vf*TA on the production of (+)-neomenthylamine. Reaction conditions (1 mL): 5 mM (−)-menthone, 2 mg/mL purified *Vf*TA, 1 mM PLP, KPB buffer (100 mM pH 7.0), 1% DMSO, in addition to 50 mM amino donor of L-Ala, isopropylamine, isobutylamine, (*S*)-2-pentanamine, (*S*)-2-hexaneamine, (*S*)-2-heptanamine, (*S*)-2-nonanamine, (*S*)-α-methylbenzylamine or benzylamine. All mixtures were shaken at 30 °C, 1000 rpm for 24 h.

To improve the efficiency of *Vf*TA-mediated reaction for (+)-neomenthylamine production, the effects of some reaction parameters were investigated, including amino donor types and equivalents, pH, temperature, and PLP dose. For transaminases, unfavorable thermodynamic equilibrium is an important bottleneck limiting the reaction (Slabu et al. 2018), and adding excessive amino donor usually can help to overcome this defect. We selected nine commonly used amino donors with different carbon chain lengths and steric hindrance sizes. Among them, *S*-MBA as an amino donor exhibited excellent catalytic efficiency for the transaminase reaction (Fig. 2).

**Fig. 3:**
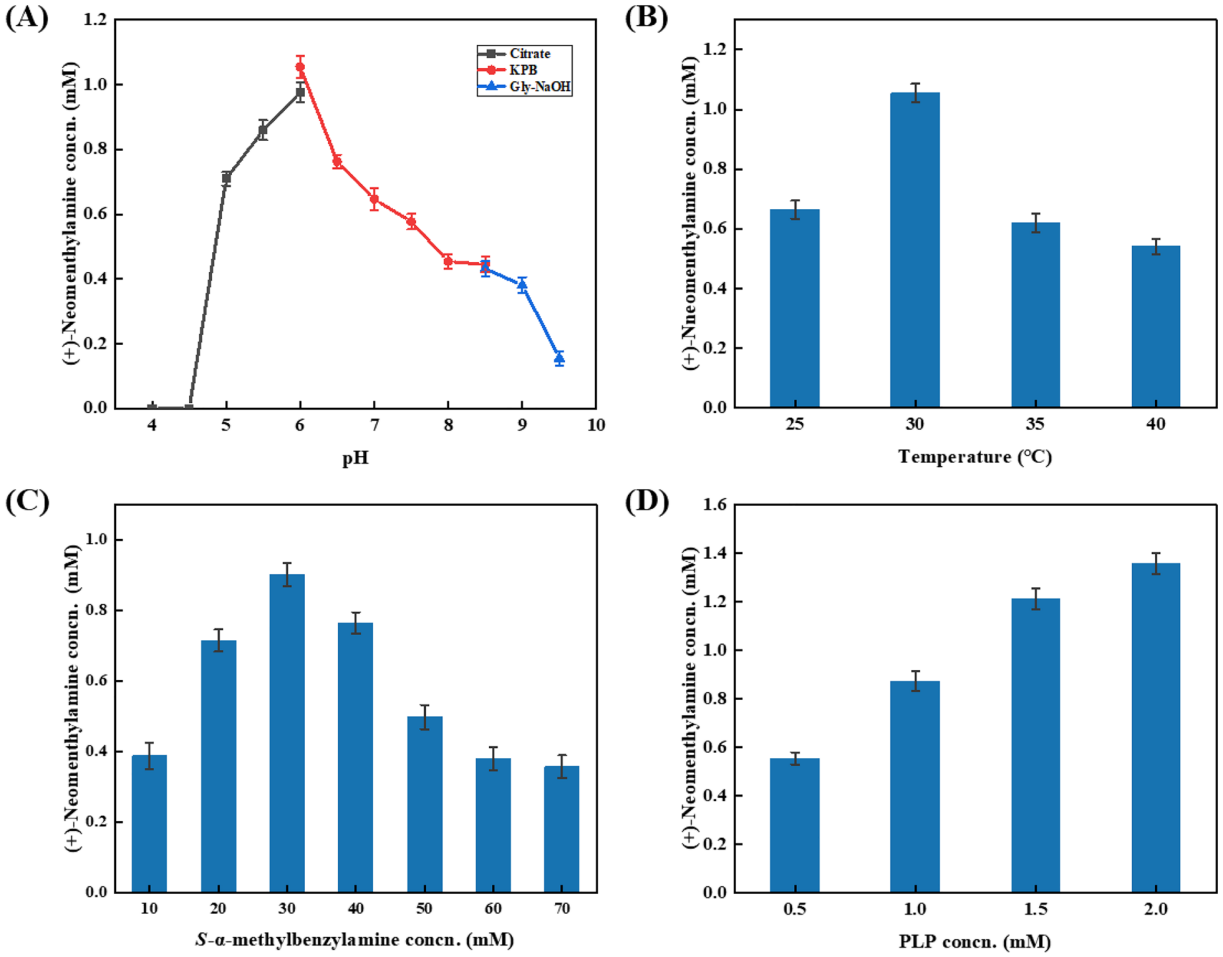
Optimization of the system parameters of *Vf*TA-catalyzed transamination. Reaction conditions (1 mL): 5 mM (−)-menthone, 50 mM (*S*)-α-methylbenzylamine, 2 mg/mL purified *Vf*TA, 1 mM PLP, KPB buffer (100 mM pH 7.0), 1% DMSO, 30 °C, 1000 rpm for 24 h, unless otherwise noted. (**A**) citrate buffer (100 mM pH 4.0–6.0) or potassium phosphate buffer (100 mM pH 6.0–8.5) or Gly-NaOH buffer (100 mM pH 8.5–9.5); (**B**) incubation temperature: 25 °C, 30 °C, 35 °C, or 40 °C; (**C**) 10–70 mM (*S*)-α-methylbenzylamine; (**D**) 0.5–2 mM PLP.

The optimal pH and temperature for the reaction were determined to be pH 6.0 (Fig. 3A) and 30 ℃ (Fig. 3B), respectively. The titer of (+)-neomenthylamine raised with the increase of amino donor equivalent, and the highest titer of (+)-neomenthylamine was observed when 60 mM of *S*-MBA was loaded (Fig. 3C). However, further increase of amino donor equivalent posed a detrimental effect on the yield of (+)-neomenthylamine (Ge et al. 2022). Besides, the dosage of cofactor PLP (pyridoxal-5'-phosphate) was also optimized, indicating that 2 mM PLP was sufficient for the single-step reaction (Fig. 3D).

### Kinetic analysis for *Vf*TA showed potential co-substrate and co-product inhibition

*Vf*TA from *V. fluvialis* JS17 belongs to the class III transaminases of the fold type I PLP family, which follows a ping-pong bi-bi reaction mechanism (Slabu et al. 2017). Amino donor *S*-MBA participates in the reaction as a co-substrate. Wild-type *Vf*TA was purified and used for determination of kinetic constants (Table 2). As a result, *Vf*TA had very limited catalytic efficiency (*k*_cat_/*K*_M_) for the substrate (−)-menthone, whereas it had remarkable higher catalytic efficiency (*k*_cat_/*K*_M_) for amino donor *S*-MBA.

**Table 2:** Kinetic constants of *Vf*TA toward substrate (−)-menthone and amino donor *S*-MBA^a^

Entry	Compound	*V*_max_ (U/mg)	*K*_M_ (mM)	*k*_cat_ (s^−1^)	*k*_cat_/*K*_M_ (s^−1^ mM^−1^)
*Vf*TA	(−)-Menthone	0.0054 ± 0.0002	5.0 ± 0.6	16.3 ± 0.0	3.3
	(*S*)-α-Methylbenzylamine	0.014 ± 0.000	0.8 ± 0.1	42.3 ± 0.0	54.4

^a^All reactions were carried out in triplicates, and the average values with standard deviation were shown. The kinetic curves of *Vf*TA for substrate and amino donor were fitted by Prism 9

In the structure of *Vf*TA (PDB: 5ZTX), cofactor PLP was covalently linked to lysine 285 through Schiff base, forming a highly conserved catalytic domain. This linkage affected the conformational stability and flexibility of the dimer interface, including the catalytic region (Additional file 1: Fig. S1). Next, we established molecular models between crystal structure and different ligands through AutoDock Vina. The docking analysis showed that the ligand *S*-MBA adopted a flat conformation within the binding pocket, while (−)-menthone faced more steric hindrance due to its inherent chair structure (Additional file 1: Fig. S2). Meanwhile, protein–ligand interaction analysis revealed that *S*-MBA had more hydrophobic interactions and hydrogen bonds with surrounding residues compared with (−)-menthone, promoting its binding to the enzyme. The π–π stacking interaction between *S*-MBA and Y150 stabilized the binding of the amino donor, playing a crucial role in activating activity (Additional file 1: Fig. S3). Notably, this situation was in accordance with the significantly lower *K*_M_ value of *Vf*TA for *S*-MBA compared to (−)-menthone, indicating a stronger affinity of *Vf*TA toward *S*-MBA.

We also investigated whether the amination of (−)-menthone by *Vf*TA was affected by substrate or product inhibition during the reaction. Using the inhibition model established based on Michaelis–Menten equation (Choi et al. 2017), the corresponding inhibition constants were calculated (Table 2). Unusually, *Vf*TA exhibited substrate inhibition and product inhibition for *S*-MBA and acetophenone, while no corresponding inhibitions were detected for (−)-menthone and (+)-neomenthylamine. Furthermore, *Vf*TA had a much lower *K*_i_ value for acetophenone compared with *S*-MBA, resulting in a ninefold difference in *K*_i_. Even when 20 mM acetophenone was added, the rate of enzymatic reaction was almost undetectable (Table 3).

**Table 3:** Inhibition constants of *Vf*TA toward *S*-MBA and acetophenone

Entry	Compound	*K*_i_ (mM)
*Vf*TA	*S*-α-Methylbenzylamine	5.8 ± 0.7
	Acetophenone	0.6 ± 0.0

^a^All reactions were carried out in triplicates, and the average values with standard deviation were shown. The kinetic curves of *Vf*TA for *S*-MBA and acetophenone were fitted by Origin 2018

### Thermostability analysis of *Vf*TA

The thermostability of *Vf*TA was explored by both melting temperature (*T*_m_) measurements and half-life (*t*_1/2_) measurements after incubation at different temperatures. The *T*_m_ value of *Vf*TA (0.5 mg/mL) in KPB buffer (100 mM, pH 6.0) was determined to be 65.6 °C (Additional file 1: Fig. S4). The deactivation rate constant (*k*_d_) and *t*_1/2_ following the first-order enzymatic inactivation kinetic equation were also determined. As shown in Table 4 and Additional file 1: Fig. S4, the *t*_1/2_ of *Vf*TA at 30 °C was close to 2 days, and even at 50 °C, it could still be maintained for 3 h. Thus, it is evident that *Vf*TA possesses commendable thermal stability.

**Table 4:** Determination of thermostability parameters of *Vf*TA

Entry	30 ℃		40 ℃		50 ℃		*T*_m_^a^
	*k*_d_/h	*t*_1/2_/h	*k*_d_/h	*t*_1/2_/h	*k*_d_/h	*t*_1/2_/h	℃
*Vf*TA	0.02	44.1	0.09	8.1	0.2	3.1	65.6 ± 0.4

^a^The melting temperature (*T*_m_) curves of *Vf*TA were fitted by Global 3 T-Ramp

### 300 mL preparative-scale bioreaction

**Fig. 4:**
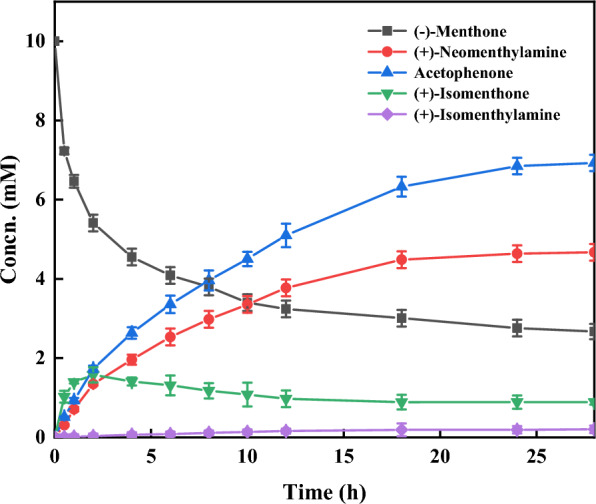
Time courses of the 300 mL preparative-scale asymmetric synthesis of (+)-neomenthylamine. Reaction condition: 10 mM (−)-menthone, 60 mM *S*-MBA, 100 g L^−1^ *Vf*TA whole cells, 2 mM PLP, KPB (100 mM pH 6.0), 30 ℃ and 200 rpm, 28 h

Finally, we performed a 300 mL preparative-scale bioreaction under optimal conditions, where the time-course analysis revealed that the yield of (+)-neomenthylamine reached a maximum 4.7 mM at 24 h approximately (Fig. 4). Surprisingly, we observed that (−)-menthone undergoes racemization in aqueous solution at the beginning of the reaction, generating a small portion of (+)-isomenthone. Subsequently, (+)-isomenthone was transaminated to produce (+)-isomenthylamine, and the side reaction reached equilibrium after 12 h. The reaction was incubated for 28 h, and after derivatization and purification, 150 mg of *N*-Boc derivatized (+)-neomenthylamine product was obtained with 33% yield. [α]_D_^23^ =  + 30 (*c* 1.7, ethyl acetate). ^1^H NMR (600 MHz, CDCl_3_): δ 4.52 (d, *J* = 8.5 Hz, 1H), 4.00–3.91 (m, 1H), 1.77 (d, *J* = 13.3 Hz, 1H), 1.74–1.68 (m, 1H), 1.68–1.58 (m, 2H), 1.37 (s, 9H), 1.34–1.28 (m, 1H), 1.00–0.87 (m, 3H), 0.85 (d, *J* = 6.7 Hz, 3H), 0.82 (d, *J* = 6.6 Hz, 3H), 0.80 (d, *J* = 6.5 Hz, 3H), 0.78–0.75 (m, 1H).

Thus, the pathway for synthesizing (+)-neomenthylamine using biocatalyst has been well established, where mild aqueous conditions are used, all the reactants used in this route [(−)-menthone, *S*-MBA, and PLP] are inexpensive, and the process generated the easily removable by-product acetophenone, with minor environmental impact. In contrast to the previously reported chemical methods, the constructed biocatalytic approach could represent an easier and greener approach for (+)-neomenthylamine synthesis. A number of strategies that may be utilized to further enhance the yield, such as continuous feeding of amino donors, or in situ removal of co-products. In addition, a significant advantage of *Vf*TA is its excellent stability. Freeze-dried *Vf*TA enzyme powder is readily available and could maintain its catalytic activity for decades when stored at 4 °C.

## Conclusion

In summary, we developed a novel and simple route to achieve the biosynthesis of (+)-neomenthylamine. By screening a number of wild-type enzymes, an ω-transaminase *Vf*TA from *Vibrio fluvialis* JS17 capable of synthesizing (+)-neomenthylamine was obtained. After systematic characterization of enzymatic properties and kinetics analysis, a 300 mL scaled-up bioreaction was conducted with considerable yield. In contrast to the previously reported chemical methods, the constructed biocatalytic approach avoided the use of expensive and poisonous catalysts, harsh operation conditions, and generation of excess wastes, thus representing an easy and green approach for (+)-neomenthylamine synthesis. More works of protein engineering and process optimization may be necessary to further improve the efficiency of biotransformation and to minimize the waste of the excessive co-substrate. The refined enzymatic route might provide new accesses to other structurally varied terpene-derived primary amines and associated optically active compounds.

### Supplementary Information

**Additional file 1: Table S1.** Amino acids sequences of enzymes for functional screening. **Figure S1.** Dimeric structure of ω-transaminase from *Vibrio fuvialis* JS17. **Figure S2.** Docking results of different ligands with *Vf*TA. **Figure S3.** Interactions between different ligands and residues. **Figure S4.** Melting temperatures of the *Vf*TA. **Figure S5.** Kinetic curves of inhibition of *Vf*TA towards *S*-MBA and acetophenone. **Figure S6.** Kinetic curves of *Vf*TA towards substrate and amino donor. **Figure. S7.** ^1^H NMR and ^13^C NMR spectra of the (+)-*N*-Boc-neomenthylamine.

## Data Availability

All data generated or analyzed during this study are included in this article and its supplementary information file.

## Abbreviations

*S*-MBA: *S*-α-Methylbenzylamine
KPB: Potassium phosphate buffer
DMSO: Dimethyl sulfoxide
D.e.: Diastereomeric excess
Concn.: Concentration
*Vf*TA: Transaminase from *Vibrio fluvialis* JS17
*Cb*FDH: Formate dehydrogenase from *Candida boidinii*
*Bm*GDH: Glucose dehydrogenase form *Bacillus megaterium*
NADH: Nicotinamide adenine dinucleotide
NADPH: Nicotinamide adenine dinucleotide phosphate
PLP: Pyridoxal-5′-phosphate
TAs: Transaminases
IREDs: Imine reductases
AmDHs: Amine dehydrogenases
RedAms: Reductive aminases
ADCLs: Aminodeoxychorismate lyases
