# Supplementary material for: Construction and optimization of a biocatalytic route for the synthesis of neomenthylamine from menthone
Source: Bioresour Bioprocess. 2023 Nov 3;10(1):75. doi: 10.1186/s40643-023-00693-w (PMC10992614; doi:10.1186/s40643-023-00693-w)
Supplement: Supplementary file 1 — Additional file 1: Table S1. Amino acids sequences of enzymes for functional screening. Figure S1. Dimeric structure of ω-transaminase from Vibrio fuvialis JS17. Figure S2. Docking results of different ligands with VfTA. Figure S3. Interactions between different ligands and residues. Figure S4. Melting temperatures of the VfTA. Figure S5. Kinetic curves of inhibition of VfTA towards S-MBA and acetophenone. Figure S6. Kinetic curves of VfTA towards substrate and amino donor. Figure. S7. 1H NMR and 13C NMR spectra of the (+)-N-Boc-neomenthylamine. [file 40643_2023_693_MOESM1_ESM.docx]

Additional file for

**Construction and Optimization of a Biocatalytic Route for the Synthesis of Neomenthylamine from Menthone**

**Hui-Jue Zhu, Jiang Pan, Chun-Xiu Li, Fei-Fei Chen* and Jian-He Xu***

Laboratory of Biocatalysis and Synthetic Biotechnology, State Key Laboratory of Bioreactor Engineering, Shanghai Collaborative Innovation Centre for Biomanufacturing, College of Biotechnology, East China University of Science and Technology, Shanghai 200237, People's Republic of China.

* Corresponding authors: Dr. Fei-Fei Chen and Prof. Jian-He Xu. E-mail: feifeichen@mail.ecust.edu.cn; jianhexu@ecust.edu.cn

**Table S1. Amino acids sequences of transaminases for functional screening.**

| **Enzyme** | **Microorganism** | **Sequence** | **Identity (%)** |
| --- | --- | --- | --- |
| *Vf*TA  (PDB:5ZTX) | *Vibrio fuvialis* JS17 | MASMTGGQQMGRGSMNKPQSWEARAETYSLYGFTDMPSLHQRGTVVVTHGEGPYIVDVNGRRYLDANSGLWNMVAGFDHKGLIDAAKAQYERFPGYHAFFGRMSDQTVMLSEKLVEVSPFDSGRVFYTNSGSEANDTMVKMLWFLHAAEGKPQKRKILTRWNAYHGVTAVSASMTGKPYNSVFGLPLPGFVHLTCPHYWRYGEEGETEEQFVARLARELEETIQREGADTIAGFFAEPVMGAGGVIPPAKGYFQAILPILRKYDIPVISDEVICGFGRTGNTWGCVTYDFTPDAIISSKNLTAGFFPMGAVILGPELSKRLETAIEAIEEFPHGFTASGHPVGCAIALKAIDVVMNEGLAENVRRLAPRFEERLKHIAERPNIGEYRGIGFMWALEAVKDKASKTPFDGNLSVSERIANTCTDLGLICRPLGQSVVLCPPFILTEAQMDEMFDKLEKALDKVFAEVAAA | 100 |
| *Pm*TA  (PDB:5G09) | *Priestia megaterium* | MSLTVQKINWEQVKEWDRKYLMRTFSTQNEYQPVPIESTEGDYLIMPDGTRLLDFFNQLYCVNLGQKNQKVNAAIKEALDRYGFVWDTYATDYKAKAAKIIIEDILGDEDWPGKVRFVSTGSEAVETALNIARLYTNRPLVVTREHDYHGWTGGAATVTRLRSYRSGLVGENSESFSAQIPGSSYNSAVLMAPSPNMFQDSDGNLLKDENGELLSVKYTRRMIENYGPEQVAAVITEVSQGAGSAMPPYEYIPQIRKMTKELGVLWINDEVLTGFGRTGKWFGYQHYGVQPDIITMGKGLSSSSLPAGAVLVSKEIAAFMDKHRWESVSTYAGHPVAMAAVCANLEVMMEENFVEQAKDSGEYIRSKLELLQEKHKSIGNFDGYGLLWIVDIVNAKTKTPYVKLDRNFTHGMNPNQIPTQIIMKKALEKGVLIGGVMPNTMRIGASLNVSRGDIDKAMDALDYALDYLESGEWQ | 23.96 |
| *Ach*TA  (WP_251865455.1) | *Achromobacter sp. Marseille-Q4962* | MSAAKLPDLSHLWMPFTANRQFKANPRLLASAKGMYYTSFDGRQILDGTAGLWCVNAGHCREEIVSAIASQAGVMDYAPGFQLGHPLAFEAATAVAGLMPQGLDRVFFTNSGSESVDTALKIALAYHRARGEAQRTRLIGRERGYHGVGFGGISVGGISPNRKTFSGALLPAVDHLPHTHSLEHNAFTRGQPEWGAHLADELERIIALHDASTIAAVIVEPMAGSTGVLVPPKGYLEKLREITARHGILLIFDEVITAYGRLGEATAAAYFGVTPDLITMAKGVSNAAVPAGAVAVRREVHDAIVNGPQGGIEFFHGYTYSAHPLAAAAVLATLDIYRREDLFARARKLSAAFEEAAHSLKGAPHVIDVRNIGLVAGIELSPREGAPGARAAEAFQKCFDTGLMVRYTGDILAVSPPLIVDENQIGQIFEGIGKVLKEVA | 30.61 |
| *ArtS*TA  (5G2P_A) | *Arthrobacter* sp. | MGLTVQKINWEQVKEWDRKYLMRTFSTQNEYQPVPIESTEGDYLITPGGTRLLDFFNQLCCVNLGQKNQKVNAAIKEALDRYGFVWDTYATDYKAKAAKIIIEDILGDEDWPGKVRFVSTGSEAVETALNIARLYTNRPLVVTREHDYHGWTGGAATVTRLRSFRSGLVGENSESFSAQIPGSSCSSAVLMAPSSNTFQDSNGNYLKDENGELLSVKYTRRMIENYGPEQVAAVITEVSQGVGSTMPPYEYVPQIRKMTKELGVLWISDEVLTGFGRTGKWFGYQHYGVQPDIITMGKGLSSSSLPAGAVVVSKEIAAFMDKHRWESVSTYAGHPVAMAAVCANLEVMMEENLVEQAKNSGEYIRSKLELLQEKHKSIGNFDGYGLLWIVDIVNAKTKTPYVKLDRNFRHGMNPNQIPTQIIMEKALEKGVLIGGAMPNTMRIGASLNVSRGDIDKAMDALDYALDYLESGEWQQS | 24.11 |
| pTA  (WP_063301853.1) | *Pseudovibrio sp.* WM33 | MDYIANLPPTHVLQEKDAAHHLHPFTDTKSLNAKGTRVITRADGVYLWDSEGNKILDGMAGLWCVNVGYGRQEIIDAVYRQMQQLPYYNTFFQSSHPPAIGLAERISSLAPDHLDHVFFAGSGSEANDTVVRMVRHYWASEGKPTKKTIISRHNAYHGSTMAGASLGGMSAMHAQGGLPIPDITHINQPYWYGEGGDMDPAAFGLMRARELEAEIDRLGEDNVAAFIGEPIQGAGGVIIPPETYWPEIQRICRERNILLIADEVICGFGRTGNWFGSQTFNFKPDLMPIAKGLSSGYLPIGAVVVSEKVAKGFIEHGGEFYHGFTYSAHPAACAAALANLDIIENERLPEKVANDTGPYLAEKWKTLGEHPLVGEARICGLVGALELSPDKARRARFEAEKGTVGTICRDHCFESGLVMRHVGDSMIISPPLVISRSEVDELIQKAHRALDLTAADVTAQSIK | 39.69 |
| *Cv*TA  (PDB:7Q9X) | *Chromobacterium violaceum* ATCC 12472 | MQKQRTTSQWRELDAAHHLHPFTDTASLNQAGARVMTRGEGVYLWDSEGNKIIDGMAGLWCVNVGYGRKDFAEAARRQMEELPFYNTFFKTTHPAVVELSSLLAEVTPAGFDRVFYTNSGSESVDTMIRMVRRYWDVQGKPEKKTLIGRWNGYHGSTIGGASLGGMKYMHEQGDLPIPGMAHIEQPWWYKHGKDMTPDEFGVVAARWLEEKILEIGADKVAAFVGEPIQGAGGVIVPPATYWPEIERICRKYDVLLVADEVICGFGRTGEWFGHQHFGFQPDLFTAAKGLSSGYLPIGAVFVGKRVAEGLIAGGDFNHGFTYSGHPVCAAVAHANVAALRDEGIVQRVKDDIGPYMQKRWRETFSRFEHVDDVRGVGMVQAFTLVKNKAKRELFPDFGEIGTLCRDIFFRNNLIMRACGDHIVSAPPLVMTRAEVDEMLAVAERCLEEFEQTLKARGLA | 40.34 |
| *At*TA  (PDB:4CE5) | *Aspergillus terreus* | ASMDKVFAGYAARQAILESTETTNPFAKGIAWVEGELVPLAEARIPLLDQGFMHSDLTYDVPSVWDGRFFRLDDHITRLEASCTKLRLRLPLPRDQVKQILVEMVAKSGIRDAFVELIVTRGLKGVRGTRPEDIVNNLYMFVQPYVWVMEPDMQRVGGSAVVARTVRRVPPGAIDPTVKNLQWGDLVRGMFEAADRGATYPFLTDGDAHLTEGSGFNIVLVKDGVLYTPDRGVLQGVTRKSVINAAEAFGIEVRVEFVPVELAYRCDEIFMCTTAGGIMPITTLDGMPVNGGQIGPITKKIWDGYWAMHYDAAYSFEIDYNERN | No significant similarity found |
| ATA-117  (PDB:5FR9) | *Arthrobacter sp.* | AFSADTPEIVYTHDTGLDYITYSDYELDPANPLAGGAAWIEGAFVPPSEARIPIFDQGFYTSDATYTTFHVWNGNAFRLGDHIERLFSNAESIRLIPPLTQDEVKEIALELVAKTELREAMVTVTITRGYSSTPFERDITKHRPQVYMSASPYQWIVPFDRIRDGVHLMVAQSVRRTPRSSIDPQVKNFQWGDLIRAIQETHARGFELPLLLDCDNLLAEGPGFNVVVIKDGVVRSPGRAALPGITRKTVLEIAESLGHEAILADITPAELYDADEVLGCSTGGGVWPFVSVDGNSISDGVPGPVTQSIIRRYWELNVEPSSLLTPVQY | No significant similarity found |

**
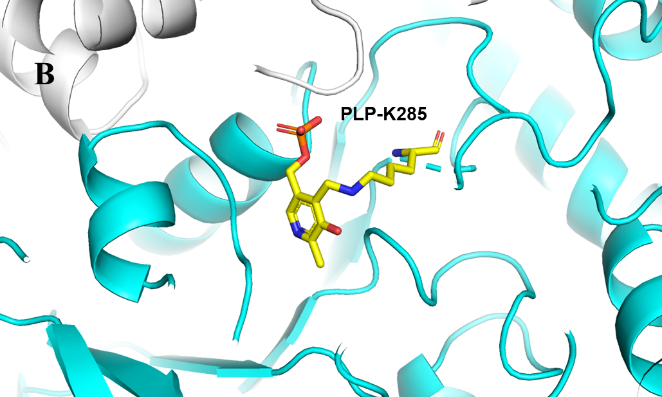

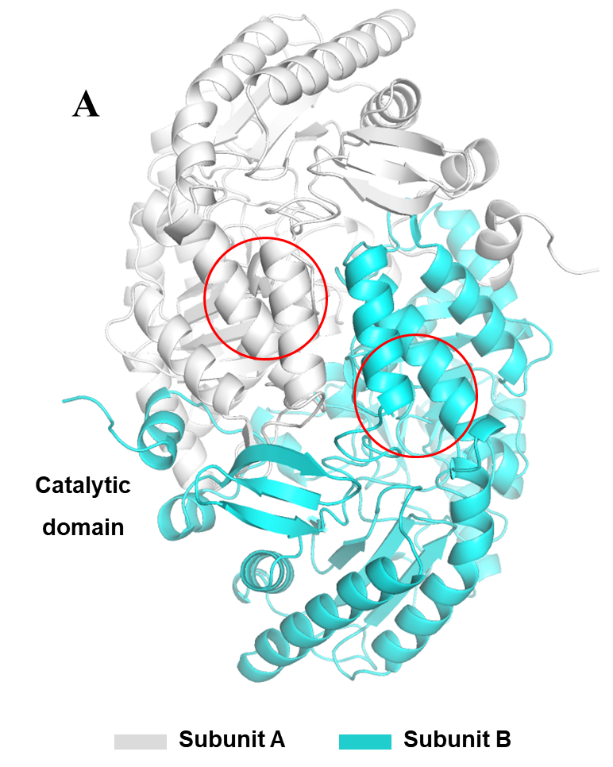
**

**Figure S1.** Dimeric structure of ω-transaminase from *Vibrio fuvialis* JS17. (A) The N-terminal helical domain in the structure. (B) PLP (pyridoxal-5'-phosphate) and K285 forming a covalent bond in the active site.


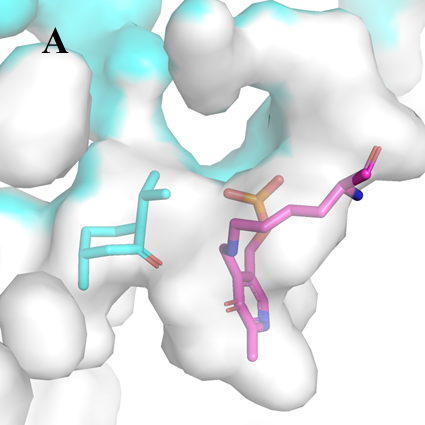

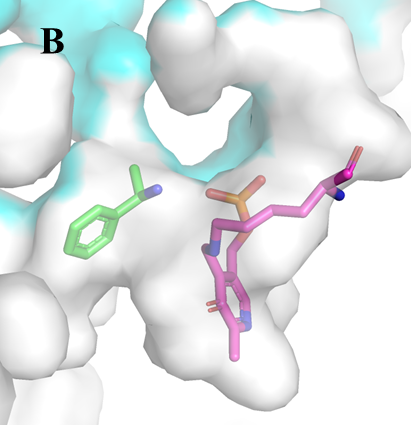


**Figure S2.** Docking results of different ligands with *Vf*TA. (A) Structure of *Vf*TA with (−)-menthone (bule). (B) Structure of *Vf*TA with *S*-MBA (green). Monomers A and B were shown in white and palecyan, respectively. The catalytic K285-PLP covalent complex is highlighted in red.


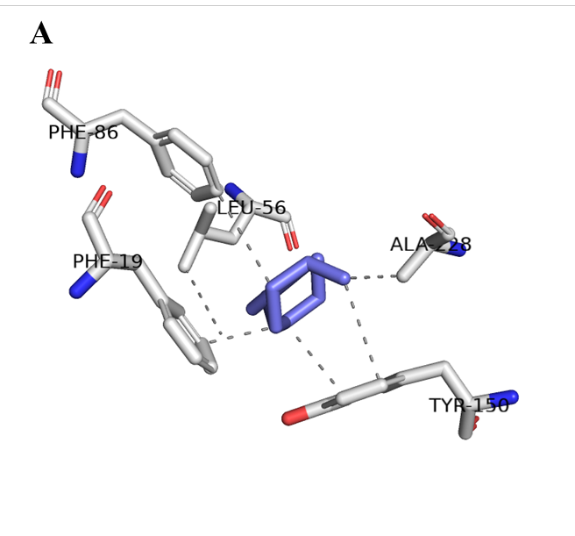

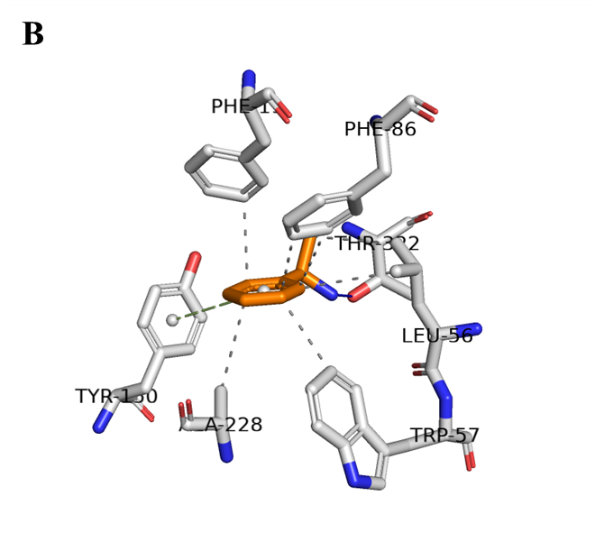


**Figure.S3.** Interactions between different ligands and residues. (A) The hydrophobic interactions between (−)-menthone (bule) and residues (white). (B) Additional π-π stacking between *S*-MBA (orange) and Y150.


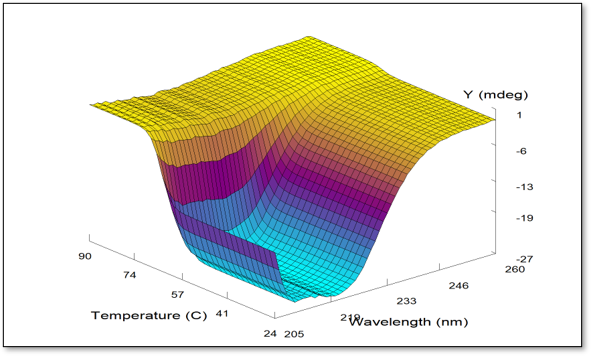


**Figure S4.** CD spectrum for determining melting temperature of *Vf*TA.


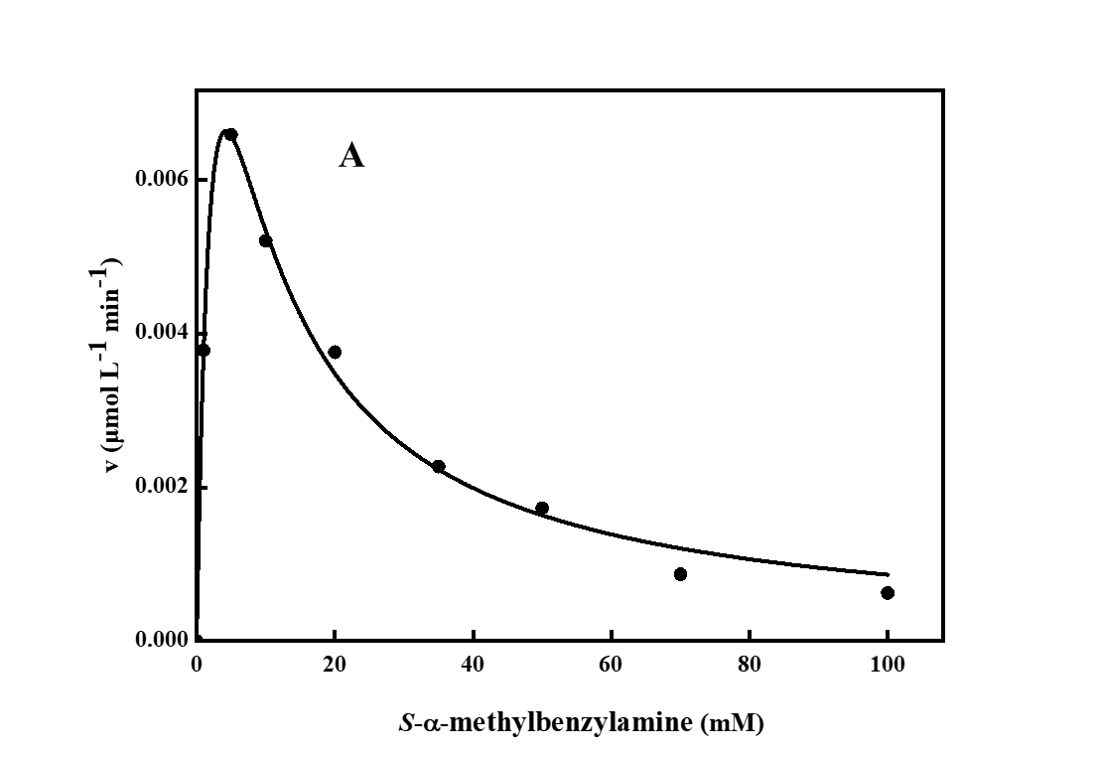

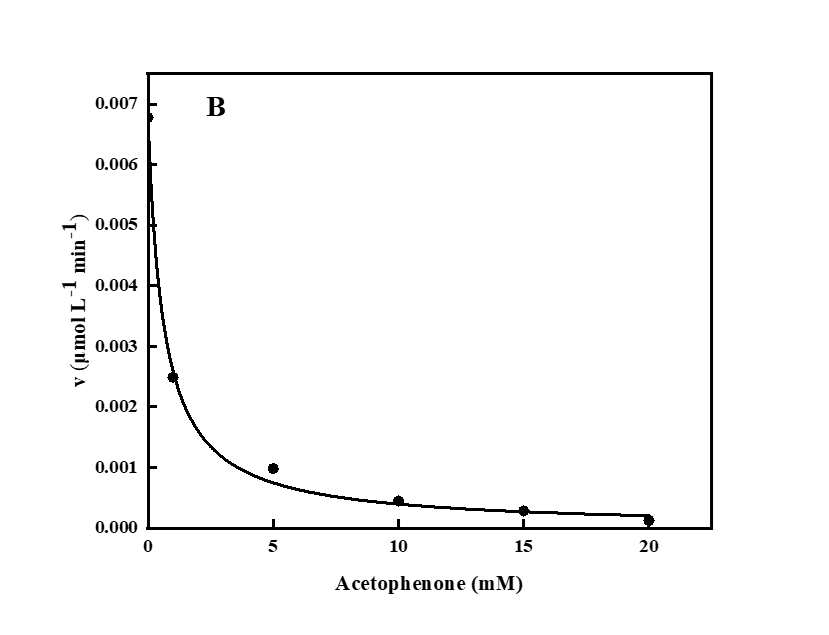


**Figure S5.** Kinetic curves of inhibition of *Vf*TA towards *S*-MBA and acetophenone.

(A) Kinetic curve for *Vf*TA towards substrate *S*-MBA; (B) Kinetic curve for *Vf*TA towards acetophenone.


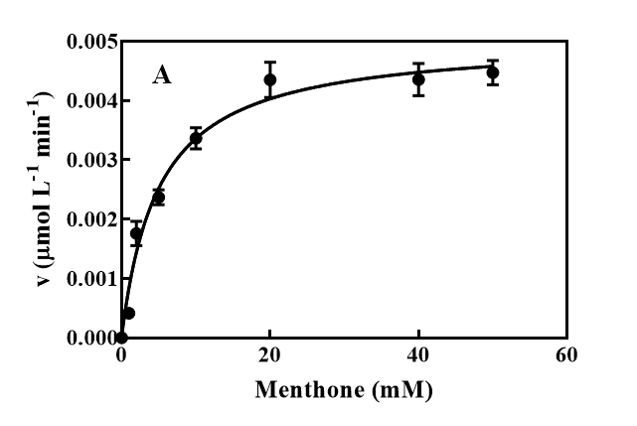

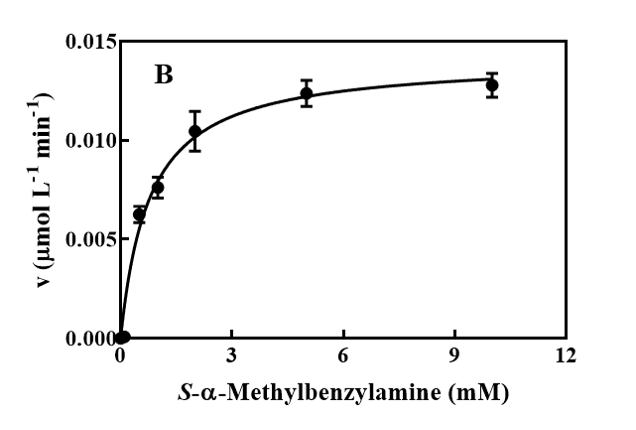


**Figure S6.** Kinetic curves of *Vf*TA towards substrate and amino donor.

1. Kinetic curve of *Vf*TA towards substrate (−)-menthone; (B) Kinetic curve of *Vf*TA towards amino donor *S*-MBA.

**Figure S7.** ^1^H NMR and ^13^C NMR spectra of the (+)-*N*-Boc-neomenthylamine.
